# Leveraging Machine Learning for Size and Shape Analysis of Nanoparticles: A Shortcut to Electron Microscopy

**DOI:** 10.1021/acs.jpcc.3c05938

**Published:** 2023-12-28

**Authors:** Christina Glaubitz, Amélie Bazzoni, Liliane Ackermann-Hirschi, Laura Baraldi, Moritz Haeffner, Roman Fortunatus, Barbara Rothen-Rutishauser, Sandor Balog, Alke Petri-Fink

**Affiliations:** † Adolphe Merkle Institute, University of Fribourg, Chemin des Verdiers 4, 1700 Fribourg, Switzerland; ‡ Department of Chemistry, Life Sciences and Environmental Sustainability, University of Parma, Parco Area delle Scienze 17/A, 43124 Parma, Italy; § Chemistry Department, University of Fribourg, Chemin du Musée 9, 1700 Fribourg, Switzerland

## Abstract

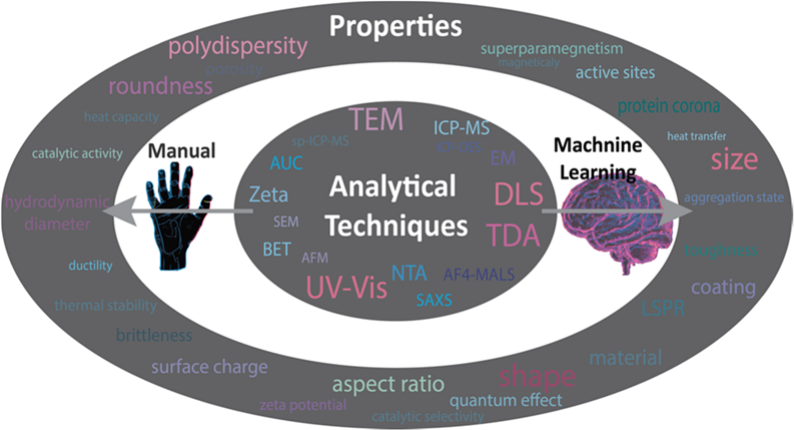

Characterizing nanoparticles (NPs) is crucial in nanoscience due to the direct influence of their physiochemical properties on their behavior. Various experimental techniques exist to analyze the size and shape of NPs, each with advantages, limitations, proneness to uncertainty, and resource requirements. One of them is electron microscopy (EM), often considered the gold standard, which offers visualization of the primary particles. However, despite its advantages, EM can be expensive, less accessible, and difficult to apply during dynamic processes. Therefore, using EM for specific experimental conditions, such as observing dynamic processes or visualizing low-contrast particles, is challenging. This study showcases the potential of machine learning in deriving EM parameters by utilizing cost-effective and dynamic techniques such as dynamic light scattering (DLS) and UV–vis spectroscopy. Our developed model successfully predicts the size and shape parameters of gold NPs based on DLS and UV–vis results. Furthermore, we demonstrate the practicality of our model in situations in which conducting EM measurements presents a challenge: Tracking in situ the synthesis of 100 nm gold NPs.

## Introduction

Nanoparticles (NPs) have become increasingly important in various fields, such as cosmetics and pharmaceutics,^1,2^ in environmental applications,^3,4^ or for harvesting energy.^5−8^ As their size approaches the nanoscale, the properties of NPs can change dramatically.^9^ Therefore, the different sizes, shapes, and surface properties of NPs can lead to unique behaviors and functions.^10−12^ With this, thorough NP characterization is essential for understanding their behavior,^13,14^ optimizing their performance,^15^ ensuring their safety,^16,17^ and constant quality.^18^

However, characterizing NPs can be challenging and time-consuming as it often necessitates using multiple techniques that complement each other, as no single analytical method offers a complete depiction of a sample.^19−21^ This poses a hurdle for researchers across various disciplines as they require extensive instrument and expertise capabilities, but access to a diverse range of techniques is often limited,^22,23^ or some techniques may need additional complex setups to measure a specific experimental condition.^24^

One of the most powerful techniques for NP characterization is electron microscopy (EM), which provides high-resolution micrographs of the sample.^21^ This allows for precise size measurements, enabling the derivation of a number-based size distribution, and can classify the particles’ shape.^25^ However, despite its effectiveness, EM is often considered expensive, difficult to operate, or hard to access.

While alternative techniques like dynamic light scattering (DLS) also provide size information—albeit with less precision—they cannot offer adequate shape information.^26^ Similarly, UV–vis spectroscopy is a technique that allows for the detection of the optical properties of NPs, which in turn provide valuable information about their size, shape, concentration, and agglomeration state.^27−29^ Compared with EM, DLS and UV–vis are considered less robust and precise techniques. However, they offer the advantages of being quicker, more cost-effective, and capable of analyzing samples in dispersion, wherefore they can monitor reactions in real time.^19−21,25^

Machine learning (ML) has emerged as a promising tool in particle characterization, as it can help to identify patterns and correlations in complex data sets. ML has been known to reduce the workflow for single measurement techniques, such as using automated image analysis software for microscopy techniques,^30−32^ or to increase the data processing speed.^33^ However, using ML to predict the outcome of one NP characterization technique based on a combination of others is a relatively new and promising approach. While there have been some successful attempts in this direction, e.g., predicting the size and shape of gold NPs (AuNPs) on simulated UV–vis data, the extrapolation to lab/experimental data was less successful.^34,35^ This may happen because simulations are evaluated on mathematical models that have a well-defined scope and limited range of validity. In other words, a model trained on simulated data can provide predictions with systematic errors when applied to data obtained via experimentation. Additionally, ML has been applied to obtain the number particle size distribution—currently only measurable using EM—based on the correlation function measured with DLS.^36−38^

Any experimental technique is based on specific physical and chemical principles, which may vary within techniques. Consequently, these techniques do not measure or quantify the same physical or chemical properties.

However, there exists a mathematical relationship—which may be either unknown or arbitrarily complex, among these sets, and ML has the potential to map this relationship. In our research, we undertake this task and demonstrate its applicability on EM. In this study, we demonstrate by using spherical AuNPs within the size range of 15–50 nm that ML can predict the outcome of EM. Here, transmission electron microscopy (TEM) was used in terms of size and shape descriptors based on the input of the economical DLS and UV–vis spectroscopy. The developed ML model offers more than just a time- and resource-saving solution. While predicting the outcome of expensive techniques like TEM in size and shape analysis can help avoid the cost of these measurements, the model’s applicability goes beyond that. In particular, the model can be helpful when TEM measurements are challenging, such as following in situ reactions. The measurement setup of a TEM makes tracking changes in real time during such reactions difficult.

To demonstrate the model’s applicability, 100 nm AuNPs were synthesized by using 20 nm-sized seeds, and the model was used to predict their size distribution and shape during the growth reaction.

## Methods

### Synthesis of AuNPs

Twenty batches of AuNPs measuring 15 nm were prepared following the Turkevich method.^39^ In this synthesis, 0.5 mM HAuCl_4_ was boiled with 1.7 mM sodium citrate for 15 min. The resulting 15 nm-sized AuNPs were then cooled to room temperature and stored at 4 °C overnight before ten batches were used as seeds to synthesize 50 nm particles. For this, the Brown method was then employed.^40,41^ In brief, a solution containing 144 mL of gold(III) chloride trihydrate (0.25 mM, HAuCl_4_·3H_2_O), the preprepared 15 nm gold seeds with an Au concentration of 0.0125 mM, and sodium citrate tribasic dihydrate (0.5 mM sodium citrate, C_6_H_5_Na_3_O_7_·2H_2_O) were stirred using a magnetic stirrer, to which 1.34 mL of 0.22 M hydroxylamine hydrochloride (NH_2_OH·HCl, ACS reagent) was added. The reaction mixture was stirred for 15 min, after which the AuNPs were purified through centrifugation at 3500 rpm for 20 min, followed by redispersion in 0.5 mM NaCit.

### Physiochemical Characterization of AuNPs

For TEM analysis, 5 μL of the AuNP dispersions was deposited onto a copper grid coated with a carbon membrane and examined using a microscope operating at 120 kV. The TEM was equipped with a CCD camera. The size (min Feret diameter and its standard deviation), as well as the shape parameters (aspect ratio, projection area, and perimeter) of the AuNPs were determined using an open-source image processing program called C_6_H_6_.^42^

To record the UV–vis extinction spectrum, a Jasco V-670 spectrophotometer was used with 10 mm path-length quartz Suprasil-grade cuvettes at 25 °C. Before measurement, all dispersions were diluted 20-fold in Milli-Q water.

DLS measurements of the 5-fold diluted AuNPs in Milli-Q water at 25 °C were performed using a 90 Plus Nanoparticle Size Analyzer (Brookhaven) and reusable plastic cuvettes.

### Building the ML Model

All key parameters extracted from UV–vis and DLS spectroscopy were collected in one data set. These parameters were input for the ML model, also called features, to predict the TEM size and shape parameters, called labels. The prediction is based on the rules learned in the ML model during the training stage.

Our model is based on a gradient-boosted decision tree (GBDT) algorithm implemented with the XGBoost library.^43^ These model types are known for their robustness with limited data, efficiency, flexibility, and relative ease of implementation and interpretation.^44−47^ The decision tree structure comprises nodes and branches, with non-leaf nodes representing attributes or questions and leaf nodes providing the label prediction. A regression tree algorithm is deployed with 500 consecutive learning cycles to improve predictive accuracy.

The final prediction of the tree is evaluated against a measured data point during each cycle. If the prediction fails to match the target value, then a new tree is constructed using this error. At the same time, the hyper-parameter tuning process was carried out using a Tree-structured Parzen estimator (TPE)^48^ with the grid given in the Supporting Information, which is implemented in the Optuna library.^49^ Hyperparameters are parameters specifically designed to configure an algorithm and are adjusted by the operator. In tree-based models, these hyperparameters encompass factors such as the maximum depth of the tree, the number of trees to grow, the number of variables considered during tree construction, the minimum number of samples on a leaf, or the fraction of observations used for building a tree.^46^

TPE is an automated algorithm that determines the optimal set of hyperparameters by mapping a response surface on the objective function of the probability of a score, in this case, the root-mean-squared error. To achieve the best predictability of the model, 5-fold stratified cross-validation and the mean absolute error as a metric were used to find the optimal parameter set. After training, the model is tested on unseen data to validate its predictive power.

**Figure 1:**
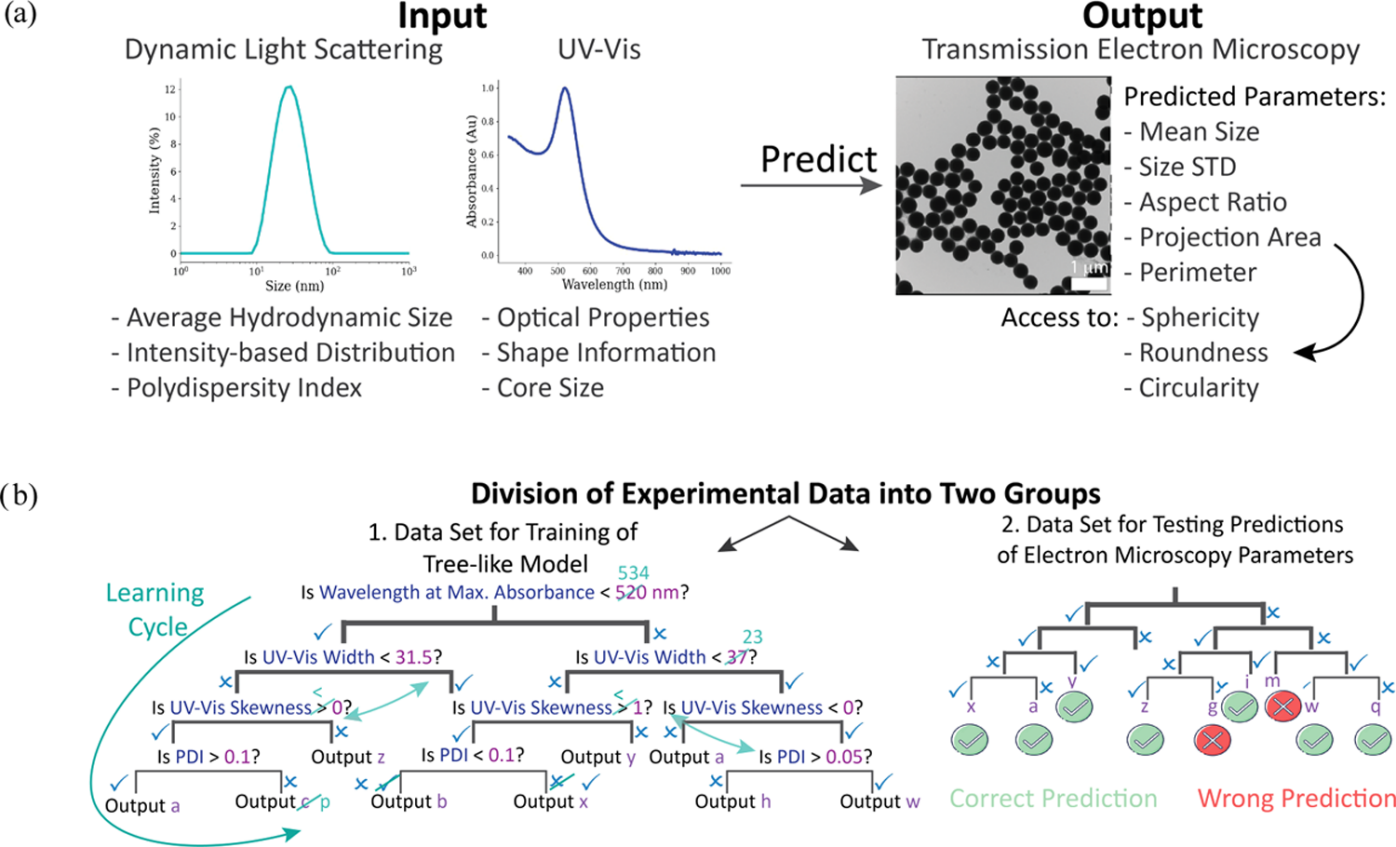
(a) ML model utilizing DLS and UV–vis spectroscopy parameters as input features to predict various size and shape parameters attainable through EM, here TEM. Additional shape indicators, such as roundness and sphericity, can be calculated with the predicted TEM parameters. (b) ML model dynamically adjusting its treelike structure during the training phase through multiple learning rounds. Decision-making rules and their corresponding values (e.g., “Is the hydrodynamic radius above 50 nm?”) of the features are rearranged to optimize the prediction of labels, in this case, the outcomes of TEM measurements. This adjustment process is based on the error in the previous model iteration. Furthermore, the optimal tree structure is evaluated, including factors such as the number of decisions the model makes before reaching a final prediction. The model demonstrating the highest predictive power is selected and subsequently tested for its accuracy in making predictions using the test data set.

The data set split into training, validation, and test sets was performed homogeneously based on the average particle ferret diameter to ensure an even data distribution. Specifically, 80% of the data were used as the training set, while 20% of the training data were reserved for the validation set. To prevent data leakage, all measurement repetitions were grouped and assigned as a single entity to the training or test set. Data leakage occurs when the model is tested on data it has already been trained on, resulting in the model strictly memorizing the data instead of learning from it.^46^ A description of the ML training process is given in Figure 1.

## Results and Discussion

### Characterization of the AuNPs

**Figure 2:**
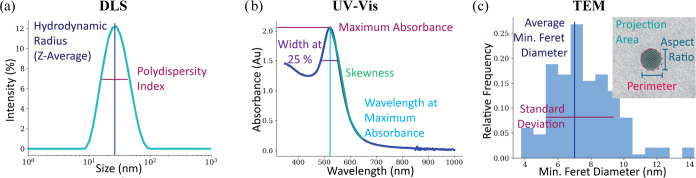
Visual description of the input and output parameters for each analytical technique. (a) Input from DLS: *Z*-average and polydispersity index. The *Z*-average is the intensity-weighted mean hydrodynamic radius, and the polydispersity index is a measurement of the homogeneity of the sample. (b) Summary of the UV–vis input parameters, including the maximum absorbance and the wavelength at which it occurs, which indicate the sample’s absorption properties. The peak width and skewness are also extracted, as they give information about the shape of the absorption peak. (c) Predicted output parameters obtainable with TEM. For size parameters, the average minimum Feret diameter and its standard deviation (STD) are predicted, and additionally, the aspect ratio, projection area, and perimeter are provided as shape descriptors.

We thoroughly characterized each AuNP dispersion with three different analytical techniques: TEM, UV–vis, and DLS. The key parameters extracted from each method are shown in Figure 2. A summary of these parameters and a brief description are listed in Table 1.

**Table 1:** Key Parameters Used to Describe the Outcomes of the Three Measurement Techniques: TEM, UV–Vis, and DLS

technique	key parameters	description	parameter extraction
TEM	(1) average min Feret diameter	minimal distance between two tangents on the opposite sides of the apparent particle outline^50^	obtained via C_6_H_6_^42^
	(2) average min Feret STD	statistical measure to indicate the degree to which the min Feret diameter of NPs in a batch differs from the average value for the group^51^	(51)
	(3) aspect ratio	ratio of a particle’s minimum Feret diameter to the maximum Feret diameter^52^	(42)
	(4) projection area	measurand for a two-dimensional area of a three-dimensional object that is projected onto an arbitrary plane^53^	obtained via C_6_H_6_^42^
	(5) perimeter	length of the particle outline^54^	obtained via C_6_H_6_^42^
UV–vis	(1) maximum absorbance	provides information on the molar concentration of the NPs through the Lambert–Beer law^29^	*y*_max_
	(2) wavelength at max absorbance	absorption of AuNPs localized surface plasmon resonance offers information about size and shape^29^	*x* = *y*_max_
	(3) peak width at 25%	full width at a three-quarter maximum of the maximum absorbance peak, indicating the sample’s homogeneity^27^ and size^28^	width = *x*_1(*y*=0.75)_ – *x*_2(*y*=0.75)_
	(4) peak skewness	measurand of the asymmetry of the maximum absorbance peak^55^	
DLS	(1) *Z*-average	intensity-weighted mean hydrodynamic diameter of a hard sphere that diffuses at the same rate as the measured NP^26^	cumulant analysis^26^
	(2) polydispersity index	size-based measurand that indicates the heterogeneity of a sample	cumulant analysis^26^

### Development of an ML Model

**Figure 3:**
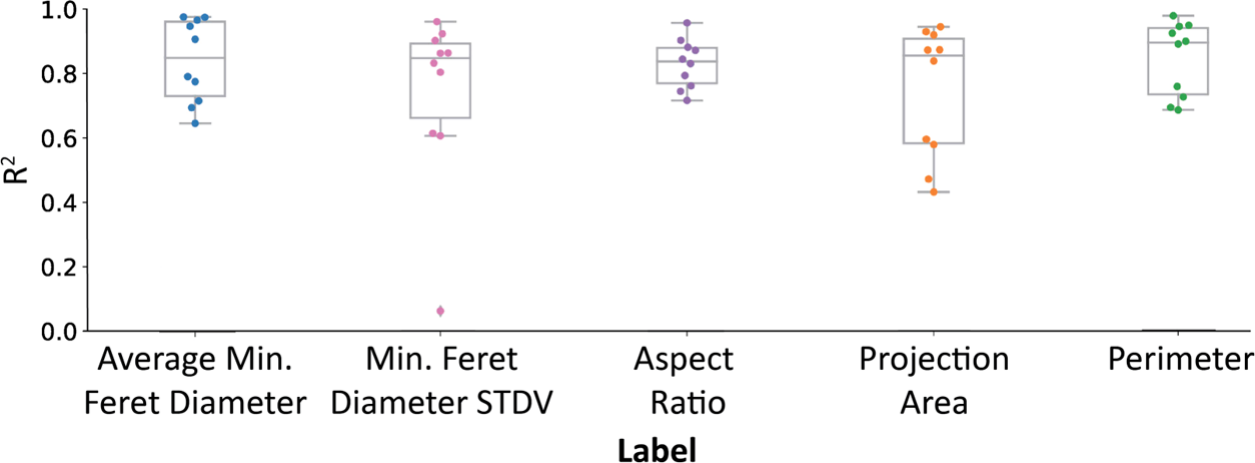
Scattered boxplots of holdout *R*^2^ scores during testing obtained with 10 independent random seeds for five labels that describe the size and shape of NPs. Those parameters are traditionally obtained by TEM but were here predicted based on DLS and UV–vis spectroscopy features.

After extracting the key parameters, we proceeded to input them into the XGBoost algorithm to construct our ML models. A total of five models were built, each corresponding to one of the following size and shape parameters: the minimum Feret diameter, standard deviation of the minimum Feret diameter, projection area, perimeter, and aspect ratio. For each parameter, we repeated the training ten times. The predictive capability of each model can be assessed by evaluating its performance on unseen data during the testing phase. One way to measure this predictive power is through the use of the *R*^2^ score, which indicates the level of agreement between the regression model and the target variable. The *R*^2^ score is a coefficient of determination, and a value of 1 signifies a perfect match between the predictions and the actual measured results.^56^ As the *R*^2^ score approaches 1, the model’s performance improves, reflecting its effectiveness in making accurate predictions. The predictive performance during testing of these models can be observed in Figure 3 for all parameters, and exemplary parity plots are shown in the Supporting Information.

The built models exhibit an average *R*^2^ score above 0.8, indicating the feasibility of predicting TEM parameters using cost-effective and easily accessible UV–vis and DLS techniques. However, the scattered boxplots reveal the presence of outliers, particularly when predicting the projection area. This shows us that (a) predicting the projection area might come with a higher uncertainty and (b) the predictive power is highly dependent on the data split into training and testing sets.

The projection area is notably sensitive to various experimental factors including illumination, magnification, and defocus. Consequently, they are highly susceptible to measurement uncertainties. De Temmerman identified the surface area as the descriptor with the highest level of uncertainty.^57^ As a result, this measurement uncertainty manifests itself in the decreased predictive power of the ML model.

The occurrence of outliers can often be attributed to the model’s sensitivity to the specific characteristics of the presented data. Therefore, it is crucial to carefully split the entire data set into training and testing data to mitigate this issue.^58^ In this study, the split was performed similarly for all models while maintaining stratification based on the average minimum Feret diameter. However, despite the stratified split, it appears that this particular stratification might not have been the most optimal choice for the projection area parameter.

### Implementation of Our Model

**Figure 4:**
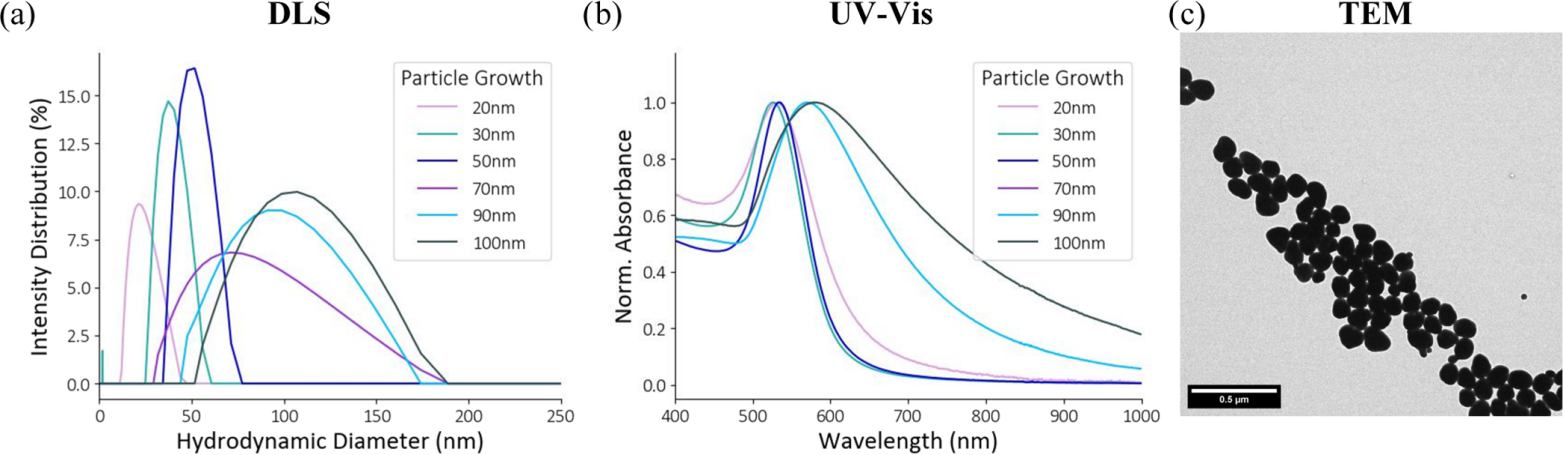
DLS (a) and UV–vis (b) to monitor the growth reaction of 20–100 nm AuNPs in situ at six different time points during the synthesis. The time points are *t* = 0 min (pink line), *t* = 10 min (teal line), *t* = 70 min (lilac line), *t* = 100 min (blue line), and *t* = 190 min (gray line). For validation, a TEM micrograph of the final particles is displayed in (c).

Monitoring reaction kinetics in real time on nanometer length scales is crucial to comprehending reaction kinetics and growth mechanisms. *Ex situ* analysis—such as EM techniques—falls short of providing the vital information needed to optimize the synthesis process since they cannot capture the development of nanostructures as it transpires in real time.^59,60^ An advantage of our model is that it can leverage DLS and UV–vis—both techniques with minimal sample preparation and measurement time needed, wherefore they come close to being in situ techniques—to predict the outcome with TEM. To demonstrate this, we tracked the growth of 20 nm AuNPs into particles with a 100 nm diameter (synthesis procedure is described in the Supporting Information). Figure 4a,b illustrates an example of following a particle growth in situ using DLS and UV–vis measurements, with micrographs of the final particles shown in Figure 4c. Table 2 summarizes all of the predicted size and shape parameters.

**Table 2:** Predicted Size and Shape Parameters Based on UV–Vis and DLS Spectroscopy, with Validation for the Time Points *t* = 0 and 190 min Using the Measured Parameters from the Synthesis Seeds and the Final Particles as well as the Predictions during the Synthesis, for Which No Control Is Possible

time point	average min Feret diameter (nm)	STD min Feret diameter (nm)	aspect ratio	perimeter (nm)	projection area (nm^2^)
predicted: *t* = 0 min	17 ± 3	3.14 ± 1	0.89 ± 0.04	57 ± 7	234 ± 52
measured: *t* = 0 min	19 ± 3		0.89 ± 0.03	54 ± 4	268 ± 77
predicted: *t* = 190 min	98 ± 5	3.75 ± 2	0.88 ± 0.07	299 ± 15	6374 ± 103
measured: *t* = 190 min	102 ± 5		0.88 ± 0.06	312 ± 9	6402 ± 939
predictions of following the synthesis in situ					
predicted: *t* = 10 min	29 ± 3	4.32 ± 2	0.89 ± 0.05	96 ± 5	673 ± 26
predicted: *t* = 40 min	45 ± 2	4.43 ± 1	0.90 ± 0.04	151 ± 16	1788 ± 110
predicted: *t* = 70 min	61 ± 3	4.46 ± 1	0.90 ± 0.03	200 ± 9	2955 ± 93
predicted: *t* = 100 min	83 ± 5	3.71 ± 2	0.90 ± 0.07	282 ± 12	5460 ± 122

When examining the starting and end point of the synthesis, which represent the points where we can validate our model, we observe a remarkable alignment between the measured and predicted values. This further emphasizes the strong predictive power exhibited by the developed models. Although we do not have control over the predicted data throughout the synthesis process, we possess a high level of confidence in the models’ ability to demonstrate significant predictive capability in those stages, as well.

Moreover, we extended the application of our model to predict the size and shape parameters of spongosomes—low-contrast particles that typically necessitate staining or cryo-TEM for analysis, further complicating their study. Remarkably, our model demonstrated strong agreement between the predicted parameters and the measured values, as evidenced in the Supporting Information.

## Conclusions

Combining orthogonal analytical techniques is essential for the comprehensive characterization of NPs. However, operating multiple techniques can be time-consuming and costly. In this work, we explore ML-based shortcuts for orthogonal techniques. We could predict the outcome of size and shape parameters, traditionally obtainable with TEM, based on DLS and UV–vis by training an ML model on different fully characterized AuNP batches with different sizes and polydispersity indices. This model can be used in laboratories with limited access to TEM or for experiments where TEM is difficult to apply. Therefore, we applied our model to follow an in situ reaction.

While this current model is trained on spherical AuNPs, we show that the development of ML for orthogonal techniques has the potential to revolutionize the field of NP characterization, making it more accessible, efficient, and cost-effective.

## Supporting Information Available

Additionally, we make several files (including primary data) available at 10.5281/zenodo.10245524. The Supporting Information is available free of charge at https://pubs.acs.org/doi/10.1021/acs.jpcc.3c05938.

Used NP batches and ML training parameters, as well as the synthesis procedure of the 100 nm AuNPs, and the extrapolation of the model (PDF)

## Abbreviations

AuNPs: gold nanoparticles
DLS: dynamic light scattering
EM: electron microscopy
ML: machine learning
NPs: nanoparticles
STD: standard deviation
TEM: transmission electron microscopy
