# Supplementary material for: Leveraging Machine Learning for Size and Shape Analysis of Nanoparticles: A Shortcut to Electron Microscopy
Source: J Phys Chem C Nanomater Interfaces. 2023 Dec 28;128(1):421–7. doi: 10.1021/acs.jpcc.3c05938 (PMC10788956; doi:10.1021/acs.jpcc.3c05938)
Supplement: Supplementary file 1 — jp3c05938_si_001.pdf [file jp3c05938_si_001.pdf]

Leveraging Machine Learning for Size and Shape Analysis of Nanoparticles: A Shortcut to Electron Microscopy

Christina Glaubitz,[a] Amélie Bazzoni,[a] Liliane Ackermann-Hirschi,[a] Laura Baraldi,[b] Moritz Haeffner,[a] Roman Fortunatus,[a] Barbara Rothen-Rutishauser,[a] Sandor Balog,[a] and Alke Petri-Fink [a,c]**

[a]Adolphe Merkle Institute, University of Fribourg, Chemin des Verdiers 4, 1700 Fribourg, Switzerland

[b]Department of Chemistry, Life Sciences and Environmental Sustainability, University of Parma, Parco Area delle Scienze 17/A, 43124, Parma, Italy

[c]Chemistry Department, University of Fribourg, Chemin du Musée 9, 1700 Fribourg, Switzerland

Supporting Information

Content

1. DATA SUMMARY 2
2. MACHINE LEARNING MODEL 5
3. REPRESENTATIVE PARITY PLOTS 7
4. 100 NM GOLD PARTICLE SYNTHESIS 8
5. INPUT DATA FOR PREDICTING THE PARTICLE SIZE AND SHAPE DURING SYNTHESIS 10
6. MODEL EXTRAPOLATION 11
REFERENCES 12

1. Data Summary

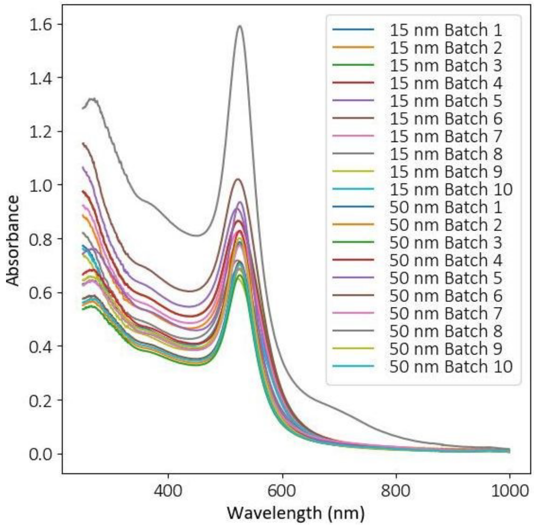

Figure S1. UV-Vis spectra of the AuNP batches.

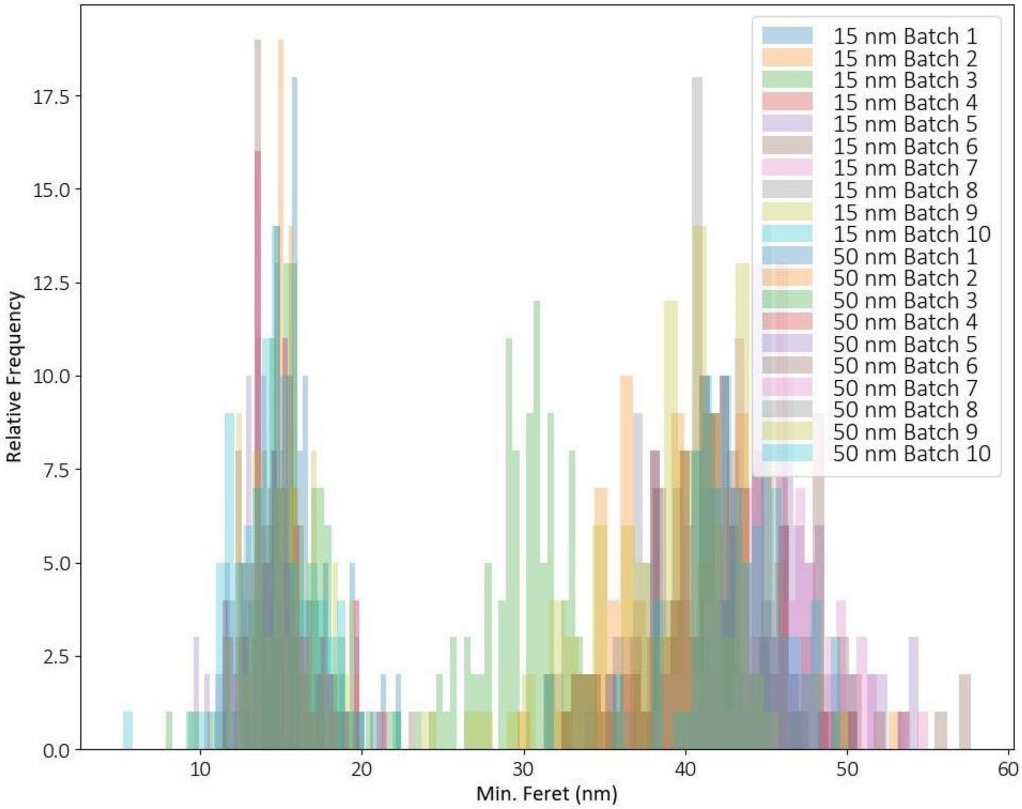

Figure S2. TEM histograms of the AuNP batches.

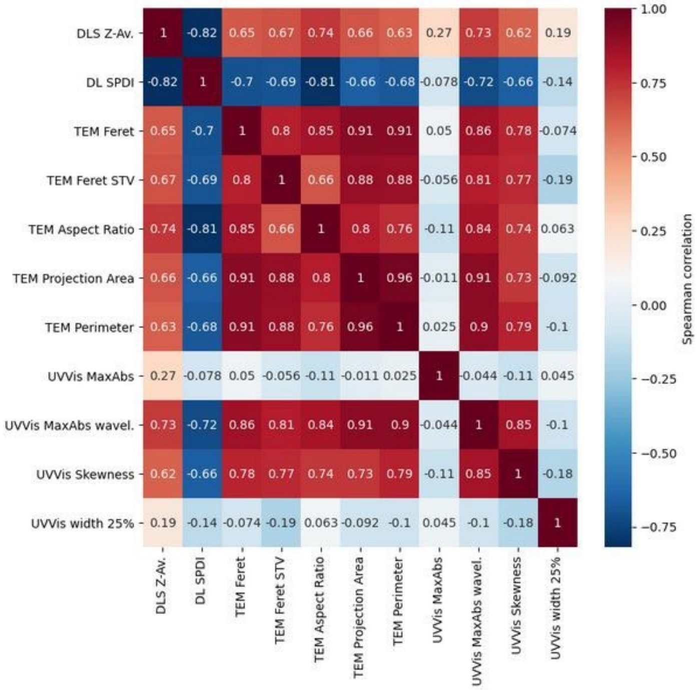

Figure S3. Spearman's rank correlation matrix of the parameters measured via DLS, UV-Vis, and TEM. A Spearman correlation of 1 signifies a perfect positive correlation, 0 implies no correlation, and -1 suggests an inverse correlation between two parameters.

Table S1. Summary of the measured parameters obtained from DLS, UV-Vis, and TEM for each particle batch

	**DLS**		**UV-Vis**				**TEM**				
**Particle Batch**	Z-Average (nm)	PDI	Max. Absorbance	Wavelength at Max. Absorbance (nm)	Peak Width at 25 % (nm)	Peak Skewness	Min. Feret Diam. (nm)	Feret SDV (nm)	Aspect Ratio	Projection Area (nm^2)	Perimeter (nm)
15 nm Batch 1	17	0.66	0.68	524	68	-0.28	17	2	0.86	260	60
15 nm Batch 2	19	0.50	0.77	524	84	-0.34	15	2	0.85	215	56
15 nm Batch 3	17	0.60	0.77	524	67	-0.33	15	2	0.85	251	61
15 nm Batch 4	18	0.58	0.86	522	84	-0.34	15	2	0.86	208	56
15 nm Batch 5	21	0.26	0.91	521	72	-0.35	14	2	0.84	165	49

15 nm Batch 6	19	0.41	1.0	522	69	-0.30	15	2	0.85	186	52
15 nm Batch 7	19	0.58	0.82	452	69	-0.32	15	2	0.83	185	52
15 nm Batch 8	20	0.47	0.71	515	72	-0.31	14	1	0.86	167	49
15 nm Batch 9	20	0.37	0.65	521	71	-0.33	15	2	0.87	200	54
15 nm Batch 10	16	0.61	0.68	524	67	-0.29	15	2	0.86	205	56
50 nm Batch 1	59	0.19	0.78	526	76	0.10	43	4	0.92	1630	155
50 nm Batch 2	50	0.24	0.68	526	70	0.90	40	5	0.94	1418	141
50 nm Batch 3	50	0.15	0.66	523	70	0.79	29	3	0.95	762	102
50 nm Batch 4	49	0.22	0.83	526	71	0.69	42	5	0.93	1510	144
50 nm Batch 5	53	0.18	0.93	528	71	0.80	44	4	0.94	1567	146
50 nm Batch 6	52	0.16	0.71	526	70	0.79	45	3	0.94	1659	148
50 nm Batch 7	50	0.17	0.77	528	69	1.43	57	3	0.94	1384	153
50 nm Batch 8	53	0.20	1.59	525	60	0.79	44	5	0.93	1188	142
50 nm Batch 9	53	0.20	0.82	523	70	0.69	38	3	0.93	1655	156
50 nm Batch 10	53	0.19	0.93	524	72	0.88	39	6	0.94	1440	144

2. Machine Learning Model

We trained a gradient-boosted decision trees as implemented in XGBoost, where we optimized the hyperparameters over the grid in Table S1 using the tree of Parzen estimator's strategy. We optimized the hyperparameters in an inner train/test split. We used the jackknife+ strategy[2] technique to obtain prediction intervals, as implemented in MAPIE,[3] considering absolute conformity scores on a validation set. For train/test splits, we stratified on the target column, which we binned in four approximately equally populated bins. To estimate robustness, we performed the workflow ten times with different random seeds.

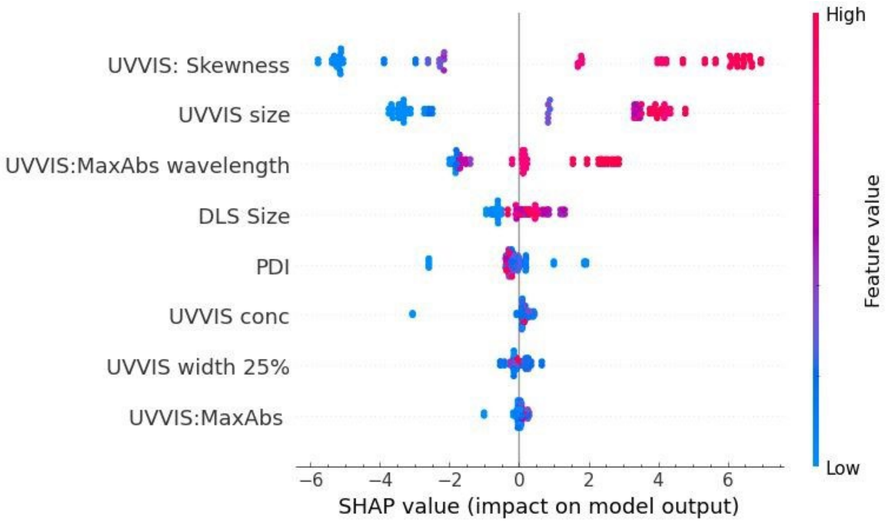

Figure S4. Summary of the SHAP analysis to determine the feature importance for the model predicting the TEM min. Feret diameter based on DLS and UV-Vis. Each point in the plot indicates a data point, and the grey vertical line indicates the average predicted Feret diameter. A negative SHAP value (shown on the abscissa) indicates a Feret diameter predicted being low with respect to the baseline, and a positive SHAP value a predicted Feret diameter being higher. The features are ranked according to their impact on the prediction, and the most important feature has the highest position.

Table S2. Hyperparameter ranges with short explanations[1] considered for the XGBoost models.

Parameter	Range	Explanation
learning rate	$1 \cdot 10^{-2}$ – 0.25 (log)	Step size at which the model weights are updated during training A high learning rate can lead to the model overshooting the minimum, making it unstable and potentially diverging from the optimal solution. A low learning rate can result in slow convergence or getting stuck in a local minimum.
λ (L2 regularization)	$1 \cdot 10^{-8}$ – 100.0 (log)	Constant that is added to the loss function. A larger value of results in a simpler model with less variance but potentially more bias, while a smaller value of lambda results more complex model with higher variance but potentially more bias.
α (L1 regularization)	$1 \cdot 10^{-8}$ – 100.0 (log)	Constant that is subtracted from the gradient of the loss function. A higher value results in a simpler model, while a lower alpha value will allow for more complex models.
subsample	0.1 – 1.0	Rows of subsamples of the training data prior to fitting a new tree
colsample_bytree	0.1 – 1.0	Subsample ratio of columns when constructing each tree. Subsampling occurs once for every tree constructed.
max_depth	1 – 9 (int)	The maximum depth of a tree is employed to regulate over-fitting because higher depth enables the model to learn relationships that are exceedingly tailored to a particular sample.
n_estimators	[7000, 15000, 20000]	Number of built trees

3. Representative Parity Plots

a)

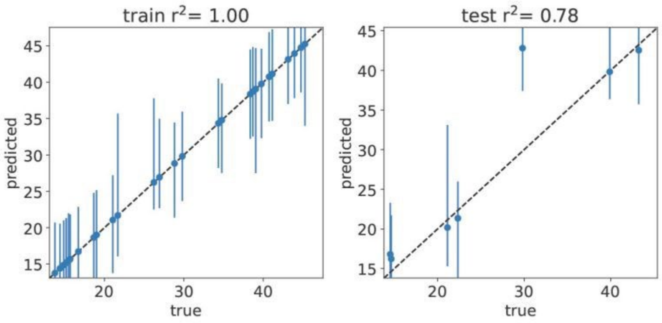

b)

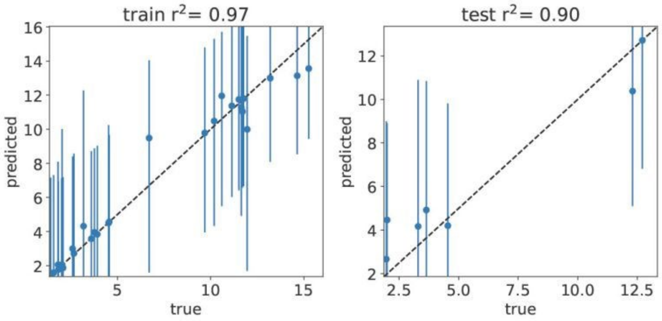

c)

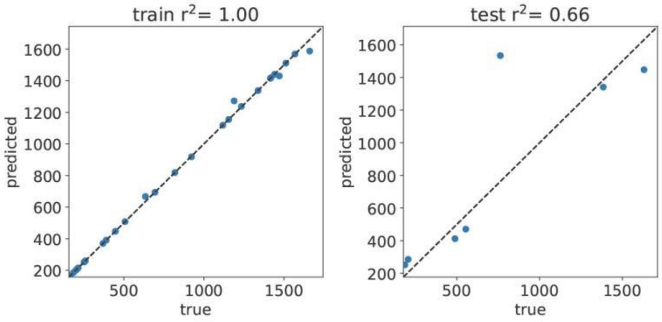

d)

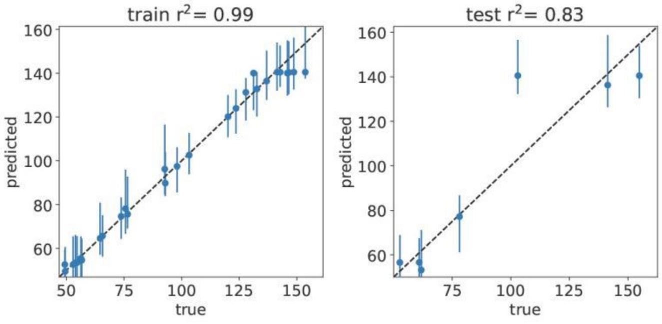

e)

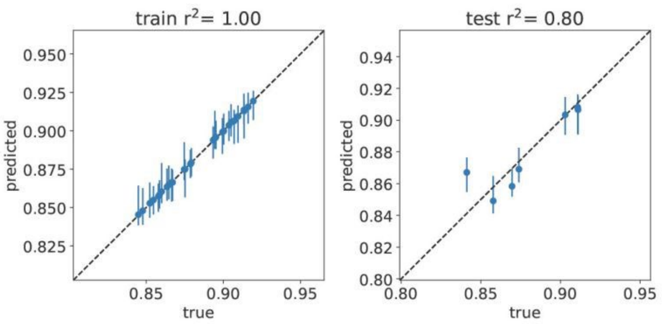

f)

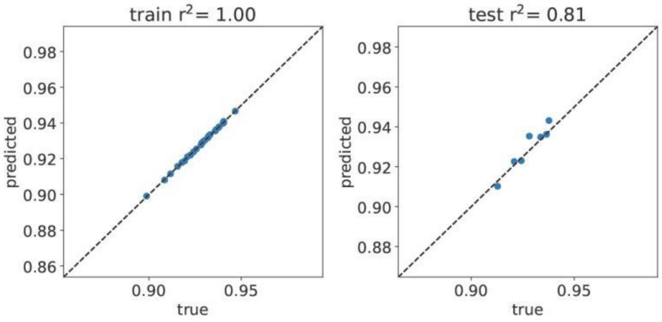

Figure S5. Representative parity plots of a) TEM average size, b) TEM size standard deviation, c) the surface area, d) particle perimeter, e) aspect ratio and f) sphericity. The black line indicates perfect overlay between predicted value (y-axis) and measured value (x-axis).

4. 100 nm Gold Particle Synthesis

First, 20 nm AuNPs were synthesised in a three-neck round-bottomed flask, by adding 1 mL of $HAuCl_4$ (25 mM) to 150 mL of sodium citrate (2.2 mM) at 100 °C. This reaction was run for 15 min until the solution turned a red-wine colour, which produced gold seeds of 10 nm and $3 \cdot 10^{-12}$ NP mL^{-1}. The solution was cooled down to 90 °C and, sequentially, 1 mL of sodium citrate (60 mM) and 1 mL of a $HAuCl_4$ solution (25 mM) were injected with a delay time of approximately 2 min, followed by a 30 min reaction period. This step was repeated one more time. The dispersion was diluted by extracting 55 ml of dispersion and adding 53 ml of MilliQ water and 2 ml of sodium citrate (60 mM) and was used for seeds to synthesise 100 nm particles. To the diluted dispersion, 1 ml of $HAuCl_4$ (25 mM) was injected, followed by a 30 min waiting period. This process was repeated until the particles reached a size of 100 nm.[4]

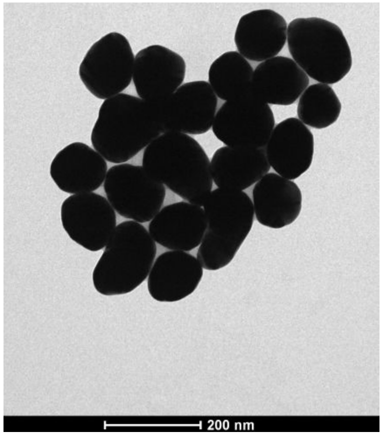

Figure S6. Representative transmission electron microscopy image for the 100 nm particles.

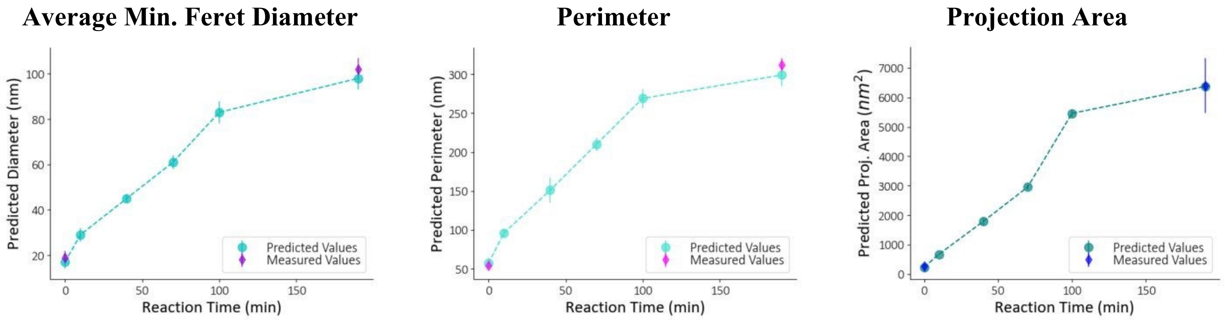

Figure S7. By leveraging ML, we can follow the growth reaction of 20 nm to 100 nm AuNPs. We use DLS and UV-Vis to monitor the reaction in situ and predict the TEM parameters (Average min. Feret Diameter, left; Perimeter, middle; Projection Area, right) during multiple timepoints. The predicated values are shown with teal colors. We validate the prediction through comparing the starting particles and the synthesized NPs with TEM, shown in violet.

5.Input Data for Predicting the Particle Size and Shape during Synthesis

Table S3. Input parameters obtained by DLS and UV-Vis analysis to predict the TEM outcome during a 100 nm AuNP synthesis.

Particle Growth	DLS Parameters		UV-Vis Parameters			
	Hydrodynamic Diameter (nm)	dispersity Index	Max. Absorbance (mAu)	Wavelength at Max. Absorbance (nm)	Skewness	Peak Width at 25 %
20 nm	24.16	0.17	0.23	532	1.19	0.27
30 nm	29.38	0.122	2.43	526	1.52	0.31
50 nm	44.51	0.10	1.10	535	1.54	0.31
70 nm	62.09	0.09	0.89	544	1.37	0.74
90 nm	85.32	0.14	0.651	572	0.49	0.26
100 nm	103.221	0.12	0.51	578	0.49	0.25

6. Model Extrapolation

Additionally, we can demonstrate that our model could even forecast the min. Feret diameter of a particle category that it was not initially trained on—spongosomes. Spongosomes are a type of lipid-polymer hybrid nanoparticles that necessitate staining or cryo-EM for visualization. Nonetheless, we managed to extrapolate our model, and it correctly predicted the min. Feret diameter for these particles. Figure S8 a) and b) show the spectra and in Table S3 the parameters that were obtained via DLS and UV-Vis that were used to predict the TEM outcome, in Figure S8 c) are displayed. This indicates the tremendous potential for incorporating additional particles into our modeling endeavors in the future.

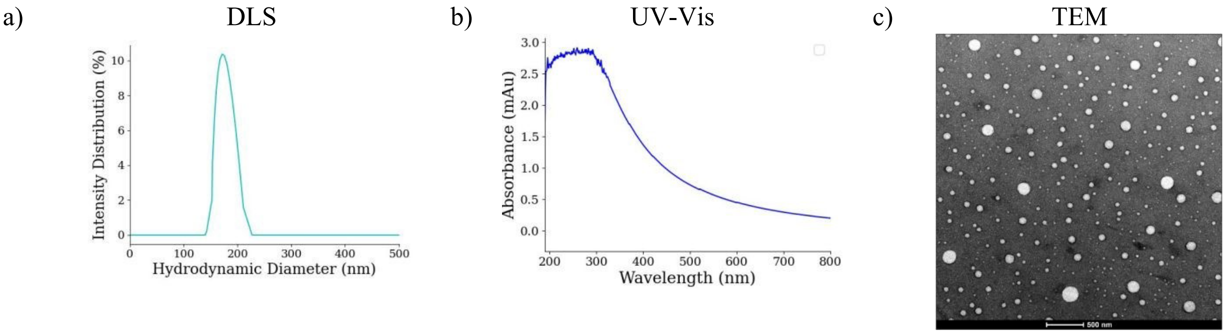

Figure S8. Panel a) depicts a TEM micrograph of the spongosomes after staining, while Panel b) illustrates the parameters obtained from DLS and UV-Vis analysis, which were utilized to predict the TEM outcome.

Table S4. Input parameters obtained by UV-Vis analysis and DLS as well as the measured and predicted TEM outcome for spongosomes.

Technique	Feature	Value	
DLS	Hydrodynamic Diameter	174 ± 1 nm	
	Polydispersity Index	0.11 ± 0.01	
UV-Vis	Peak Width at 25 %	0.27	
	Peak Skewness	1.35	
TEM	Feature	Measured Value	Predicted Value
	Average Min. Feret Diameter	149 nm	156 ± 10 nm
	Average Min. Feret STD	85 nm	51 ± 5 nm
	Aspect Ratio	0.91 ± 0.03	0.92 ± 0.02
	Projection Area	12246 ± 1476 nm^2	11029 ± 2804 nm^2
	Perimeter	364 ± 39 nm	329 ± 79 nm

References

1. Chen, T.; Guestrin, C., XGBoost: A Scalable Tree Boosting System. In *22nd SIGKDD Conference on Knowledge Discovery and Data Mining*, 2016.

2. Barber, R. F.; Candes, E. J.; Ramdas, A.; Tibshirani, R. J., Predictive inference with the jackknife+. *arXiv preprint arXiv* **2020,** *Arxiv-1905.02928*

3. Taquet, V.; Blot, V.; Morzadec, T.; Lacombe, L.; Brunel, N., Parameters or Privacy: A Provable Tradeoff Between Overparameterization and Membership Inference. *arXiv preprint arXiv* **2022,** *arXiv: Arxiv-2207.12274 2022*

4. Bastús; G, N.; Comenge, J.; Puntes, V., Kinetically controlled seeded growth synthesis of citrate-stabilized gold nanoparticles of up to 200 nm: size focusing versus Ostwald ripening *Langmuir* **2011,** *27* (17), 11098-105.
